# Screening of bacteria-binding peptides and one-pot ZnO surface modification for bacterial cell entrapment†

**DOI:** 10.1039/c7ra12302g

**Published:** 2018-02-26

**Authors:** Masayoshi Tanaka, Ilva Hanun Harlisa, Yuta Takahashi, Natasha Agustin Ikhsan, Mina Okochi

**Affiliations:** Department of Chemical Science and Engineering, School of Materials and Chemical Technology, Tokyo Institute of Technology 2-12-1, O-okayama, Meguro-ku Tokyo 152-8552 Japan okochi.m.aa@m.titech.ac.jp

## Abstract

Short functional peptides are promising materials for use as targeting recognition probes. Toll-like receptor 4 (TLR4) plays an essential role in pathogen recognition and in activation of innate immunity. Here, the TLR4 amino acid sequence was used to screen for bacterial cell binding peptides using a peptide array. Several octamer peptides, including GRHIFWRR, demonstrated binding to *Escherichia coli* as well as lipopolysaccharides. Linking this peptide with the ZnO-binding peptide HKVAPR, creates a bi-functional peptide capable of one-step ZnO surface modification for bacterial cell entrapment. Ten-fold increase in entrapment of *E. coli* was observed using the bi-functional peptide. The screened peptides and the simple strategy for nanomaterial surface functionalization can be employed for various biotechnological applications including bacterial cell entrapment onto ZnO surfaces.

## Abstract 2

Linking the screened bacteria-binding peptide with the ZnO-binding peptide HKVAPR, created a bifunctional peptide capable of one-step simple ZnO surface modification and of bacterial cell entrapment.

## Graphical abstract

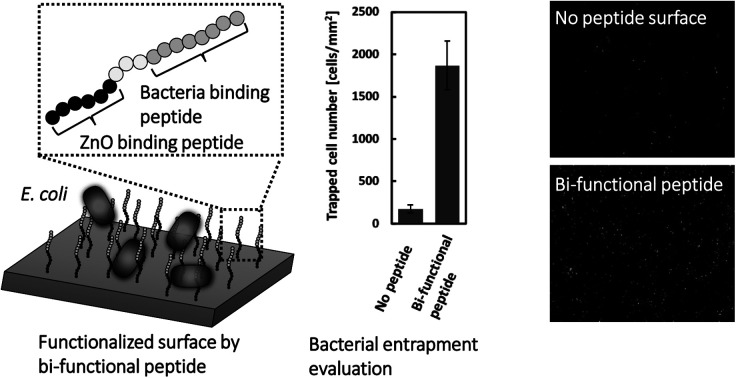

## Introduction

The introduction of desired properties onto material surfaces is one of the most important techniques for realizing various bionanotechnological applications. Short peptides, derived easily by combination of the 20 natural amino acids, are promising molecules for material surface modification as they exhibit specific recognition of material surfaces. For example, amino acids with carboxylate groups bind preferentially to titanium and silicon owing to their strong electrostatic interactions with the charged surface.^1,2^ The strong interaction between gold surfaces and tryptophan is considered to be derived from the planar structure of the amino acid with π-electron bonds characterizing its aromatic ring.^3–5^ Various peptides with these unique characteristics enabling them to bind to specific materials are used as non-covalently bound linkers for material surface modification.^6,7^

In addition to their usage for material surface modifications, peptides can bind to various targets including bacterial cells. Because of increasing worldwide concerns about pathogenic bacteria, particularly drug-resistant bacteria, bacterial recognition probes are gaining attention as tools for microbial detection.^8–12^ Peptides are promising probes as they are relatively stable and function in ligand–receptor interaction. The SPOT-synthesis technique, a method for customized peptide synthesis on a cellulose membrane established by Frank,^13^ has been adopted for the screening of functional peptides.^14,15^ In particular, the technique is useful for identifying the active site in functional proteins, owing to self-defined peptide library construction.^16,17^

Toll-like receptors (TLRs) are a class of proteins with an indispensable role in the immune system. This group of membrane bound-receptors is responsible for recognizing pathogen-associated molecular patterns^18^ and activation of innate immunity.^19^ Specifically, TLR4 recognizes lipopolysaccharides (LPS, endotoxin), eliciting signaling and inducing immune responses against invading bacteria.^20^

ZnO is receiving increasing attention because of its anti-microbial activity and its applicability for the synthesis of various nanostructures (*e.g.*, particulates and wires).^21–23^ For example, continuous mechanical cell lysis and biosensing were developed using bare ZnO nanowires.^24,25^ In this study, we conducted array-based screening of bacteria-binding peptides designed around the amino acid sequence of TLR4. We also demonstrated bacterial capturing on the ZnO surface by linking a bacteria-binding peptide to a modular peptide with affinity to ZnO.^26^ Surface functionalization is achieved *via* a one-step process, namely incubation with the peptide solution. The simple ZnO surface functionalization technique demonstrated herein could be utilized for future ZnO-based biotechnological applications, including bioseparation and biosensing.

## Results and discussion

### Array-based screening using the amino acid sequence of TLR4

To screen for bacteria-binding peptides, an 8-mer peptide library derived from the 839 amino acid sequence of human TLR4 was synthesized.^14,15^ The array was used for the binding assay with bacterial cells (*Escherichia coli*, 2 × 10^9^ cell per mL) and various peptide candidates were identified (ESI Fig. 1†). To re-evaluate the binding ability of these peptide candidates, peptide arrays comprising the 60 highest binding peptide sequences from the first screening were constructed and used for the next binding assay. Table 1 lists the top 10 bacteria-binding peptides; they exhibited a high proportion of arginine (R) and lysine (K) residues, high pI, positive charge, and low GRAVY value (hydrophilic). These results are in agreement with previous research because bacterial binding to TLR4 has been suggested to occur *via* electrostatic interactions between positively charged regions in TLR4 and negatively charged regions in the LPS.^27^ Interestingly, the top 10 peptide sequences were present in three regions of TLR4 : GRHIFWRRLRKALL (aa 802–816), KLKSLKRLTFTS (aa 348–360), and RLHKLTLRNN (aa 226–236). KLKSLKRLTFTS and RLHKLTLRNN are located in the leucine-rich repeat domain, which is thought to be responsible for TLR molecule recognition.^14,15^ Interestingly, GRHIFWRRLRKALL was also capable of binding to the bacterial cell, even though the peptide was in the cytoplasmic region. This might be ascribed to the presence of the LPS-binding motif (BBXB; B is basic amino acid and X is hydrophobic amino acid)^28^ within its sequence, even if the region seems inconsistent with the LPS-binding site of TLR4. Based on bacteria-binding results, the three linear peptides (p402:GRHIFWRR, p175:KLKSLKRL, and p114:RLHKLTLR) showing the highest fluorescent intensity in each region were subjected to further investigation.

**Table 1:** A list of peptides showing high binding to *E. coli* cells^a^

Peptide no.	Sequence	Intensity (10^6^ AU)	pI	GRAVY	Charge
p402	GRHIFWRR	3.00 ± 0.59	12.3	−1.337	+4
p404	FWRRLRKA	2.90 ± 0.43	12.3	−1.238	+4
p405	RRLRKALL	2.76 ± 0.53	12.3	−0.525	+4
p403	HIFWRRLR	2.73 ± 0.57	12.3	−0.812	+4
p175	KLKSLKRL	2.68 ± 0.34	11.3	−0.700	+4
p176	KSLKRLTF	2.40 ± 0.35	11.2	−0.425	+3
p114	RLHKLTLR	2.39 ± 0.42	12.0	−0.675	+4
p177	LKRLTFTS	1.89 ± 0.60	11.0	−0.025	+2
p115	HKLTLRNN	1.87 ± 0.55	11.0	−1.462	+3
p174	TLKLKSLK	1.81 ± 0.42	10.0	−0.225	+3

a The binding affinity of each peptide was determined by quantitative analysis using ImageQuant software for peptide array images. Average values (±SD) are based on triplicate peptide spots. The isoelectric point (pI) and the grand average of the hydropathy value (GRAVY) were analyzed using the ProtParam tool in ExPASy (http://web.expasy.org/protparam/).

### Dot blot analysis

**Fig. 1:**
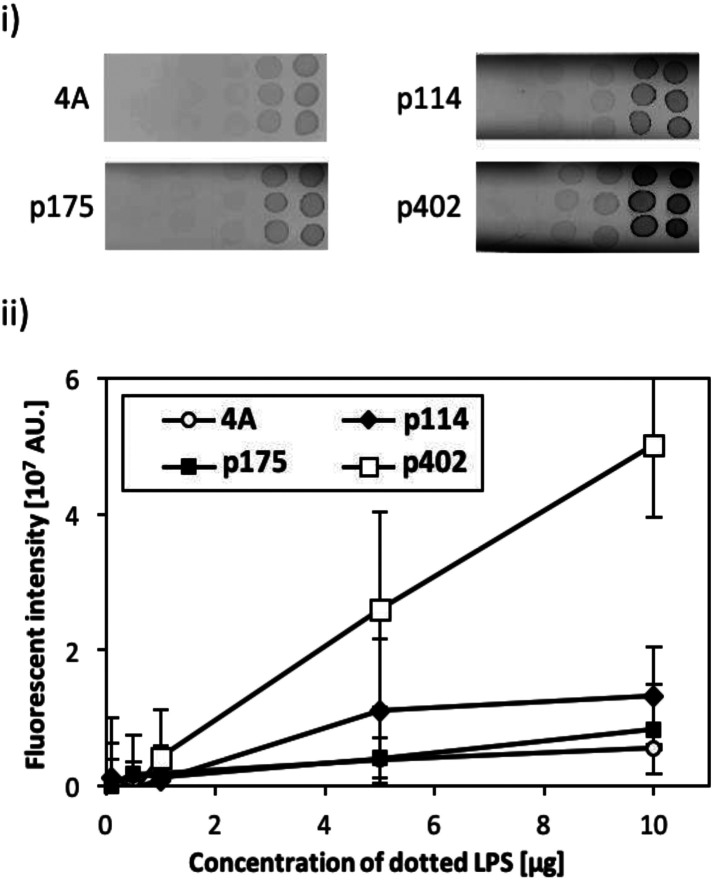
Evaluation of peptide binding to LPS by dot blot assay. (i) Fluorescent image of LPS (0.1, 0.5, 1, 5, and 10 mg) dotted strips following binding with fluorescently tagged peptides (p114, p175, and p402). A quadruple alanine peptide (4A) was used as the negative control. (ii) Quantitative evaluation of fluorescent intensity obtained from each peptide on the LPS dotted membrane strip. Error bars represent the standard deviation of three sample measurements.

The binding activity of the screened peptide candidates (p114, p175, and p402) was investigated by LPS dot blot analysis (Fig. 1). Whereas the negative control and other two candidate peptides (p114 and p175) showed negligible binding activity, peptide GRHIFWRR (p402), showed considerable binding to LPS. Hence, as peptides of the same length revealed different binding to LPS, we concluded that function did not derive simply from the number of amino acids. Accordingly, the unique binding property of each amino acid appears to result from a combination of their physicochemical properties and sequences. As LPS is the major component of the outer membrane of Gram-negative bacteria, peptide candidate p402 could bind to LPS as well as Gram-negative bacteria. On the contrary, the other two peptides could bind to bacterial cells but not to LPS, implying that they might target other cell membrane components such as membrane proteins.

### Evaluation of the binding efficiency of a bi-functional peptide on ZnO NPs

**Fig. 2:**
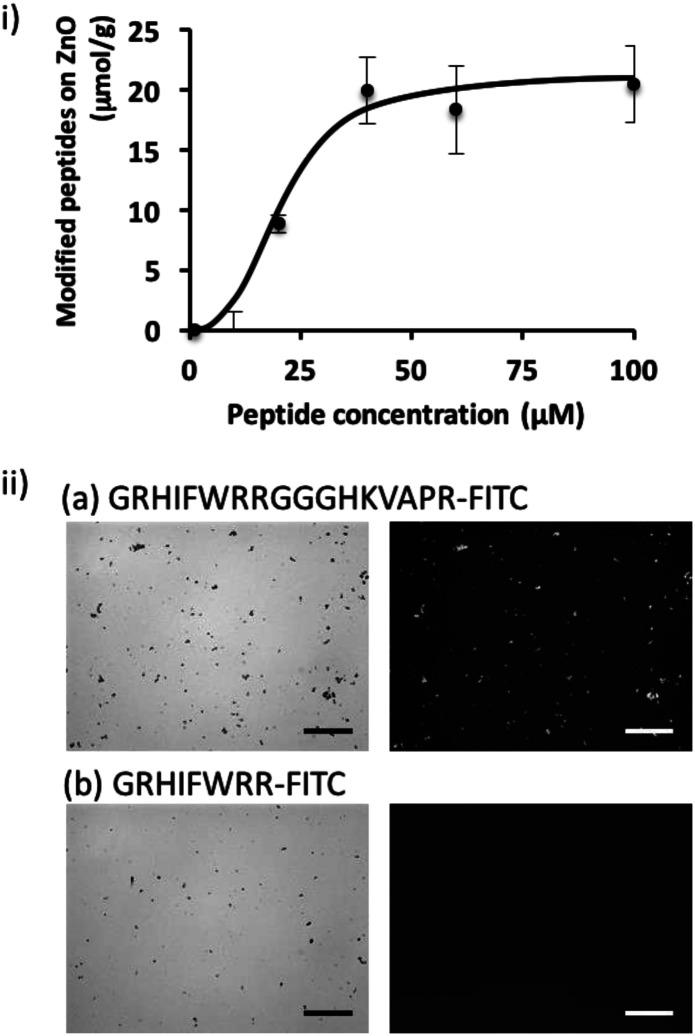
Evaluation of the binding efficiency of the FITC-labeled bi-functional peptide onto ZnO NPs. (i) ZnO NPs were modified with different concentrations of peptides from 0 to 100 μM. Based on the fluorescence quantification of the washed solution, binding efficiency was investigated. (ii) Representative phase-contrast light microscopic images (left) and fluorescence micrographs (right) of ZnO particles bound to 100 μM GRHIFWRRGGGHKVAPR-FITC (a) and GRHIFWRR-FITC (b), respectively. Scale bars indicate 20 μm.

To shed further light on the potential of the screened peptide, we investigated the functionalization of nanomaterial using a simple process. ZnO-based nanomaterials have recently received significant attention for the development of biosensors and photocatalytic anti-microbial materials,^21–23^ and introducing bacteria-binding to a material surface would be highly valuable for ZnO-based biotechnological applications. Here, we demonstrated the functionalization of a ZnO surface using a bi-functional peptide (GRHIFWRRGGGHKVAPR) consisting of the screened peptide bound to bacteria and LPS (p402:GRHIFWRR), a ZnO binding peptide (HKVAPR), and a linker peptide (GGG).^26^ First, the optimization of peptide concentration was investigated. As shown in Fig. 2; at peptide concentrations exceeding 50 μM, modification efficiency seemed to be saturated as 20 μmol g^−1^. Modification using the bi-functional peptide was observed also under the microscope. As shown in Fig. 2ii, fluorescence from the fluorescein isothiocyanate (FITC)-labeled bi-functional peptide coincided with sites of ZnO NPs visualized by phase-contrast. At the same time, FITC-labeled bacteria-binding peptide revealed negligible binding to ZnO NPs. It should be noted that the intensity of the fluorescently labeled peptide seemed to be consistently stronger for large particles and saturated for ZnO particles at this size and condition. Therefore, the investigated peptides seemed to be homogeneously distributed on the ZnO surface. These data clearly confirmed the binding property of the ZnO-binding peptide HKVAPR when fused with the bacteria-binding peptide (GRHIFWRR).

### *E. coli* entrapment on the ZnO surface using a bi-functional peptide with roles in bacteria and ZnO binding

**Fig. 3:**
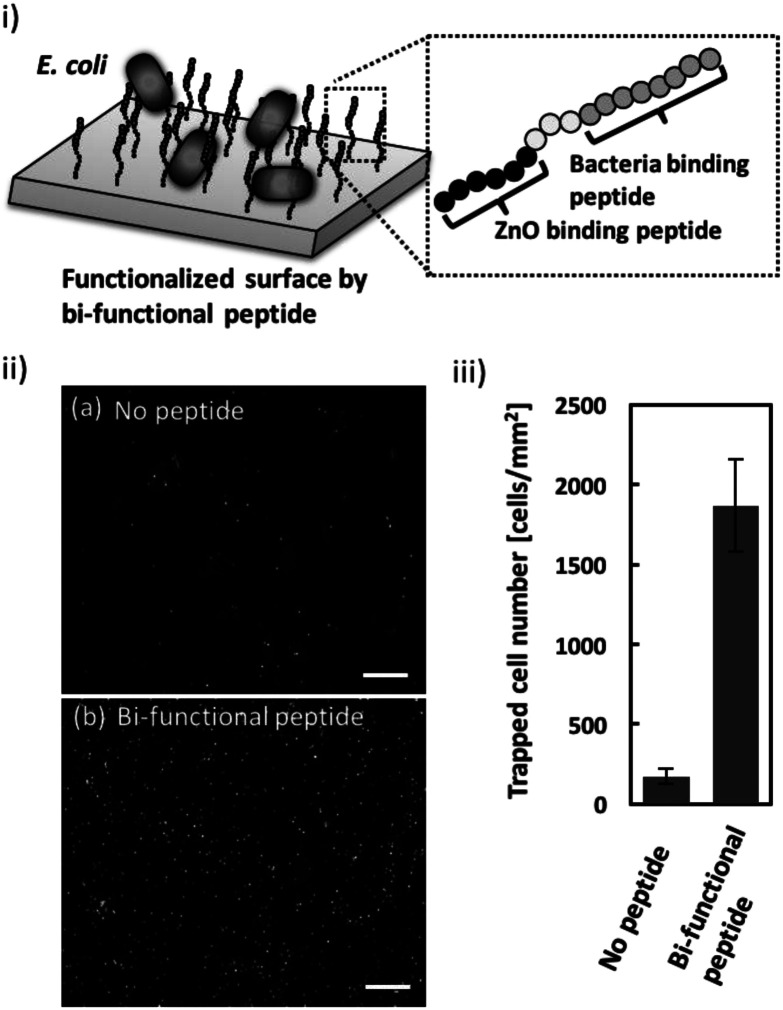
Evaluation of *E. coli* cell capture on ZnO NPs modified with a bi-functional peptide possessing *E. coli*- and ZnO-binding properties *via* a simple one-step procedure. (i) Schematic image of *E. coli* cell capture on ZnO NPs modified with bi-functional molecules. (ii) Representative fluorescence microscopy image of *E. coli* captured on ZnO NPs (a) without the peptide or (b) with the bi-functional peptide. (iii) Evaluation of the number of cells captured onto the peptide-modified ZnO NPs. Average values were obtained from three independent repeated assays. Error bars represent standard deviations. Scale bars indicate 50 μm.

Once the ZnO-binding property of the bi-functional peptide (GRHIFWRRGGGHKVAPR) was confirmed, bacterial cell capture on the peptide-modified ZnO surface was investigated (Fig. 3i). Fig. 3ii clearly shows the fluorescent signals derived from the stained cells bound to the peptide-modified ZnO, whereas unmodified ZnO rarely exhibited any fluorescence. In addition, quantitative evaluation based on the fluorescent images, revealed that binding efficiency increased approximately 10-fold following peptide modification (Fig. 3iii). These data indicate that the screened peptide possesses bacteria-binding function even when the molecule is fused to the ZnO-binding peptide and modified onto a nanomaterial surface. Furthermore, it should be noted that the ZnO surface modification was conducted using a single step; *i.e.*, incubation with the peptide solution. In this study, peptide functionalization on the ZnO surface and bacterial entrapment were conducted. However, the present conditions would benefit from further optimization of peptide sequence (*e.g.*, length of linker sequence) and peptide molecule density based on a detailed analysis of material surface properties; this would allow for the development of more effective bacterial cell entrapment tools.

## Conclusions

In conclusion, a peptide array technique was applied for the screening of bacteria-binding peptides based on the sequence of TLR4, a receptor that recognizes LPS within Gram-negative bacterial cells. Among several candidates, the GRHIFWRR peptide showed relatively high binding to LPS. The fusion peptide (GRHIFWRRGGGHKVAPR) consisting of the above peptide, linker (GGG), and ZnO-binding peptide (HKVAPR), revealed ZnO surface modification potential using a single-step method for bacterial entrapment. This simple strategy for nanomaterial surface functionalization using peptide bi-functionality can be expanded to other nanomaterials for various biotechnological applications, including biosensors.

## Materials and methods

### Screening of *E. coli*-binding peptides on the peptide array synthesized by spot technology

The peptide array was synthesized using a peptide auto-spotter (ResPep SL; Intavis AG, Köln, Germany) as reported previously.^14,15,29^ A cellulose membrane (grade 542; Whatman, Maidstone, UK) was activated using β-alanine as the N-terminal basal spacer. Activated Fmoc amino acids (0.5 M) were spotted on the membrane, using a peptide auto-spotter (MultiPep RSi; Intavis) according to the manufacturer's instructions and in all cases double couplings were applied with some modifications.^15,30^ After addition of the first residue, Acp(6) were used as additional spacers between the peptides and the cellulose membrane and the remaining amino groups were blocked with 4% acetic anhydride. For each elongation step, the membrane was deprotected with 20% piperidine in *N*,*N*′-dimethylformamide and subsequently washed thoroughly with *N*,*N*′-dimethylformamide followed by methanol. After the final deprotection, side-chain-protecting groups were removed with a solution of water, triisopropylsilane, and trifluoroacetic acid in a volume ratio of 2 : 3 : 95 for 3 h. Finally, the membrane was washed thoroughly with diethyl ether, methanol, phosphate-buffered saline (PBS, pH 7.4) and applied for binding assay. For the first screening, an 8-mer peptide library containing sequences derived from the 839 amino acid-long human TLR4 (gene ID: 7099) was synthesized. *E. coli* (BW25113) was cultured in Tryptic Soy Broth at 37 °C. In a typical binding assay experiment, 40 mL of PBS containing *E. coli* at a concentration of 2 × 10^9^ cell per mL was used. The synthesized peptide array was soaked in the *E. coli* cell suspension and incubated at 25 °C for 5 h. When screening for *E. coli*-binding peptides using the peptide array, a relatively longer incubation time was chosen, allowing for the isolation of a wide range of weak to strong binders. Subsequently, the peptide array was washed with PBS three times, stained by SYTO®9 (final concentration 1 μM) for 30 min, and washed with PBS three times. The membranes were scanned using a 494 nm excitation and 520 nm emission filter pair (Typhoon FLA 9500; GE Healthcare; Little Chalfont, UK) and were quantified using ImageQuant (GE Healthcare).

### Dot blot analysis for the evaluation of LPS binding

A polyvinylidene fluoride (PVDF) membrane was soaked in methanol, and then washed with PBS two times. After drying, 2 μL of LPS solution (0.1, 0.5, 1, 5, and 10 mg) diluted in 65% ethanol was spotted on PVDF strips and allowed to dry. The strips were blocked using 1% bovine serum albumin in PBS at 25 °C for 1 h and washed subsequently in PBS three times. The strips were incubated for 1 h 25 °C in a FITC-labeled peptide solution (final concentration 1 μM) diluted in PBS. All FITC-labeled peptides (p114, p175, p402, and AAAA; purity > 90%) were purchased from Sigma-Aldrich (Tokyo, Japan). After incubation, strips were washed three times in PBS-Tween 20 (0.05%), scanned, and quantified as above.

### ZnO surface modification using a bi-functional peptide

A bi-functional fusion peptide (GRHIFWRRGGGHKVAPR) was synthesized, purified with high-performance liquid chromatography (purity > 85%), and confirmed by mass spectrometry. Using ZnO particles (400 μg mL^−1^), ZnO surface modification efficiency was evaluated in the presence of different FITC-labeled peptide concentrations (from 0 to 100 μM). After 1 h of incubation at 25 °C, the particles were washed with PBS five times. Fluorescence intensity of all washed solutions was measured by a plate reader (PowerScan 4; DS Pharma Biomedical Co., Ltd., Osaka, Japan). Images of peptide-modified ZnO and *E. coli* capture were taken using a fluorescent microscope (DM6B; Leica, Tokyo, Japan).

### Entrapment of *E. coli* cells using a bi-functional peptide

Entrapment of *E. coli* cells on a peptide-modified ZnO surface was investigated. To fabricate the ZnO surface, ZnO particles (average diameter 25 nm) in milliQ (1 mg mL^−1^) were added to a silicon rubber-encircled hole (diameter 3 mm) attached on a glass slide. By drying up the aqueous solution, a white layer was observed indicating that a ZnO surface was fabricated onto the glass slide. After washing the surface by PBS two times, a bi-functional peptide (GRHIFWRRGGGHKVAPR, 100 μM) was added and incubated for 2 h at 25 °C. Then, the surface was washed with PBS two times. Prior to the binding assay, *E. coli* cells were stained with SYTO®9 (150 nM) for 30 min and washed twice with PBS. The stained cells (1.0 × 10^5^ cells, 10 μL) were spotted onto the peptide-modified ZnO surface and incubated for 2 h. The incubation time was shorter during the screening process to eliminate non-specific binding to ZnO surface. Following five washes with PBS, the binding of peptide-modified ZnO and *E. coli* was observed using a DM6B fluorescent microscope (Leica). Bound cells were counted manually from five randomly selected areas on the images. As a negative control, ZnO particles without peptide modification were evaluated for this assay.

## Conflicts of interest

The authors declare no competing financial interest.

## Supplementary Material

RA-008-C7RA12302G-s001
