# Supplementary material for: Screening of bacteria-binding peptides and one-pot ZnO surface modification for bacterial cell entrapment
Source: RSC Adv. 2018 Feb 26;8(16):8795–9. doi: 10.1039/c7ra12302g (PMC9078527; doi:10.1039/c7ra12302g)
Supplement: RA-008-C7RA12302G-s001 [file RA-008-C7RA12302G-s001.pdf]

Electronic Supplementary Material (ESI) for RSC Advances.
This journal is © The Royal Society of Chemistry 2018

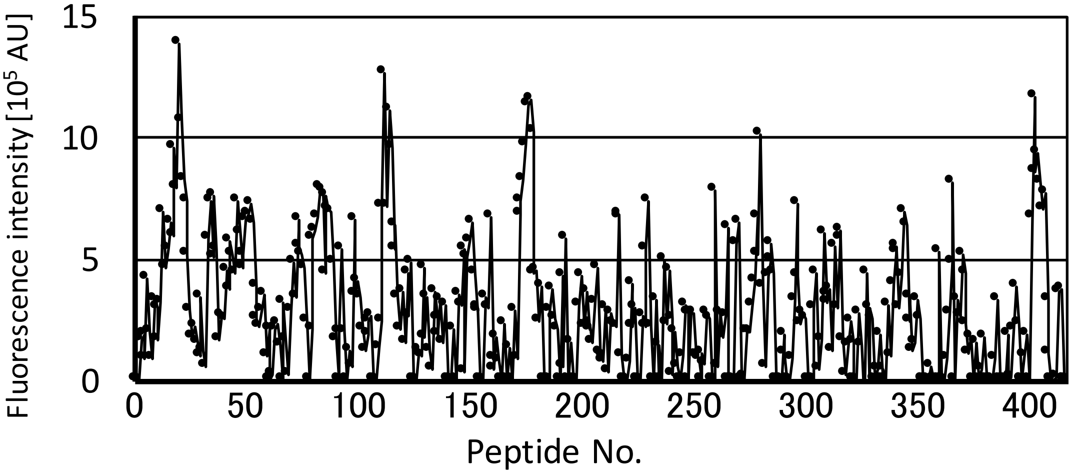

Supplemental Figure 1

Screening for *E. coli* binding peptides using a peptide array comprised of a peptide library designed based on the amino acid sequence of the TLR4 protein.

Fluorescent intensity profile of each peptide spot following the binding assay with *E. coli* cells; the fluorescent intensity in the absence of *E. coli* cells was subtracted from each sample.

Supplemental Fig. 1 Okochi et al.
